# A tool to enhance antimicrobial stewardship using similarity networks to identify antimicrobial resistance patterns across farms

**DOI:** 10.1038/s41598-023-29980-4

**Published:** 2023-02-20

**Authors:** Cecilia Aguilar-Vega, Caterina Scoglio, María J. Clavijo, Rebecca Robbins, Locke Karriker, Xin Liu, Beatriz Martínez-López

**Affiliations:** 1 Center for Animal Disease Modeling and Surveillance (CADMS), Department of Medicine and Epidemiology, School of Veterinary Medicine, University of California, Davis, CA USA; 2 Animal Health Department, Faculty of Veterinary Medicine, VISAVET Health Surveillance Centre, Complutense University of Madrid, Madrid, Spain; 3 Department of Electrical and Computer Engineering, Kansas State University, Manhattan, KS USA; 4 Department of Veterinary Diagnostic and Production Animal Medicine, Iowa State University, Ames, IA USA; 5 Pig Improvement Company (PIC), Hendersonville, TN USA; 6 Computer Science Department, University of California, Davis, CA USA

**Keywords:** Antimicrobial resistance, Infectious diseases

## Abstract

Antimicrobial resistance (AMR) is one of the major challenges of the century and should be addressed with a One Health approach. This study aimed to develop a tool that can provide a better understanding of AMR patterns and improve management practices in swine production systems to reduce its spread between farms. We generated similarity networks based on the phenotypic AMR pattern for each farm with information on important bacterial pathogens for swine farming based on the Euclidean distance. We included seven pathogens: *Actinobacillus suis*, *Bordetella bronchiseptica*, *Escherichia coli*, *Glaesserella parasuis*, *Pasteurella multocida*, *Salmonella* spp., and *Streptococcus suis*; and up to seventeen antibiotics from ten classes. A threshold criterion was developed to reduce the density of the networks and generate communities based on their AMR profiles. A total of 479 farms were included in the study although not all bacteria information was available on each farm. We observed significant differences in the morphology, number of nodes and characteristics of pathogen networks, as well as in the number of communities and susceptibility profiles of the pathogens to different antimicrobial drugs. The methodology presented here could be a useful tool to improve health management, biosecurity measures and prioritize interventions to reduce AMR spread in swine farming.

## Introduction

Antimicrobial resistance (AMR) refers to the ability of bacteria, viruses, fungi, and parasites to grow and reproduce in the presence of a given antimicrobial^1^. Antimicrobial resistance in bacteria is a great health and economic concern and one of the biggest challenges of the twenty-first century as demonstrated by the Global Action Plan on Antimicrobial Resistance supported by the World Health Organization (WHO), World Organization for Animal Health (WOAH), and the Food and Agriculture Organization of the United Nations (FAO)^2,3^. From the human perspective, 700,000 deaths every year are attributed to AMR, and only in the United States of America, at least 35,900 deaths were accredited to antibiotic-resistant bacteria and fungi each year^4,5^. From the animal perspective, loss of antimicrobial effectiveness in livestock leads to a reduction in animal welfare and production indices, as well as an increase in production and treatment costs^6,7^. This can lead to an inevitable rise in the final prices of animal products that has a negative impact on their affordability for the general public.

AMR is a complex problem, impacting human, animal and environmental health since resistance genes can disseminate to different habitats. Hence, it needs to be addressed using holistic, multi-scale methods (i.e., bacteria-, animal-, farm-, system-level) and with a One Health approach^1,8,9^. In this study, we focused on AMR in bacteria causing disease in the swine industry. Bacteria can be resistant to an antimicrobial because of an intrinsic characteristic of the species (innate resistance), or the resistance could be acquired. It is widely accepted that one main driver of acquired resistance is the selective pressure derived from antimicrobial usage, although the AMR problem is a complex one and many factors are involved. Excessive use and misuse of antibiotics either in humans or livestock could increase AMR in bacteria^2,7^. Swine production is generally characterized by being an intensive production system with high densities of animals, therefore, infectious diseases are more challenging to control even under high biosecurity conditions in case of presence or introduction^6^.

Here, we designed a framework to detect and assess similarities in phenotypic antimicrobial resistance status of farms for different pathogens based on the generation of monoplex (or single-layer) and multiplex (or multilayer) similarity networks. The overarching aim of this work is to generate a tool that could help improve management and clinical practices in large production systems to reduce the impact of AMR and minimize its spread within and between farms. In that sense, we propose some theoretical interventions for when complete data is available.

## Methods

### Data

Data from a large swine production system in the US was obtained with information on 710 animal holdings that included sow farms and finishers, although there was no information about the type of farm production for all of them. Sensitive information, such as farm names, owners, and flow was anonymized to preserve confidentiality. At each farm, we gathered information about the isolated bacterial species tested (henceforth pathogen/s). For this study, we used a total of seven pathogens: *Actinobacillus suis*, *Bordetella bronchiseptica*, *Escherichia coli*, *Glaesserella parasuis*, *Pasteurella multocida*, *Salmonella* spp., and *Streptococcus suis*. For each pathogen, we obtained the panel of antimicrobials tested by the laboratories, with its corresponding minimum inhibitory concentrations (MIC) values for the phenotypic AMR. The interpretation of the MIC value into “susceptible”, “intermediate” or “resistant” categories were primarily based on the clinical breakpoints provided by the Clinical and Laboratory Standards Institute´s (CLSI) veterinary guidelines, which are based on worldwide expert consensus^10^. When clinical breakpoints were not available by the CLSI guidelines, we used the harmonized laboratory criterion which is based on their professional experience. When interpretation for the results was missing for a given pathogen, the antimicrobial was excluded for further analysis. Category data were binarized considering resistant and intermediate as a resistant status (i.e. “1”) and susceptible as “0”. Antibiotics that were tested in less than half of the farms for a given pathogen were also excluded for subsequent analysis (Supplementary Fig. S1). In general, ten types of antibiotic classes were tested for phenotypic resistance: penicillins (ampicillin and penicillin), lincosamides (clindamycin), aminoglycosides (gentamicin, neomycin), amphenicols (florfenicol), sulfonamides (sulphadimethoxine, trimethoprim/sulphamethoxazole), fluoroquinolones (enrofloxacin), cephalosporins (ceftiofur), pleuromutilin (tiamulin), macrolides (tilmicosin, tylosin tartrate, tulathromycin), and tetracyclines (tetracycline, chlortetracycline, oxytetracycline).

The date of the reception by the laboratory was used for temporal assignment. Data ranged from the last quarter of 2018 to the end of 2021. For a given pathogen, the majority of farms had only information about one isolate for the entire study period, hence not enough information was available to conduct the analyses by year.

### Generation of monoplex networks

Monoplex networks, also known as single-layer networks, can be mathematically defined as a graph *G* = (*V*, *E*), where |*V|* is the vertex of the network, and |*E*| the edges or links representing the connections between nodes^11,12^. In this case, the nodes represented the farms where the phenotypic resistance for a set of antimicrobials of a given pathogen was tested. Edges were defined as the similarity between two farms based on the resistance pattern of a pathogen. The resistance pattern of a bacteria in a farm *F* can be defined as a vector: *F* = [*f*_*1*_, *f*_*2*_, …, *f*_*n*_], where* f* is the susceptibility status of the farm to a given antimicrobial, and *n* is the number of tested antimicrobials for a given pathogen. From this resistance pattern, we obtained the overall resistance status of a farm *F*: *RS*_*F*_ (Eq. 1). For farms with more than one isolate per pathogen, the mean susceptibility was used in the analysis.

1 $${\text{RS}}_{\text{F}}\text{=}\sqrt{\sum_{{\text{i}}= \text{1} }^{\text{n}}{{\text{f}}}_{\text{i}}^{ \, {2}}}$$

However, for a given pathogen, the resistance pattern was defined as a matrix: *P* = (*m* x *n*), where *m* is the number of farms tested for AMR for a given pathogen, and *n* the number of tested antimicrobials. To obtain the similarity of resistance patterns between farms, we first calculated the pairwise Euclidean distance^13^ (Eq. 2). The distance measure was subsequently scaled to a maximum of 1 by dividing by the maximum value, and the similarity was obtained by subtracting the distance from 1. This similarity ranged from 0 to 1, being 1 identical resistance pattern, and 0 complete dissimilarity between two given vector farms (*F* and *G*).

2 $$d\left(F, G\right)=\sqrt{\sum_{i=1}^{n}{\left({f}_{i}-{g}_{i}\right)}^{2}}$$

To overcome the problem of missing data in the resistance pattern of farms and similarity measures, we used missing data imputation^14^. Missing values for the resistance to an antibiotic were replaced by the average of the available information in other farms as a proxy of the probability of resistance in a farm with missing information.

Given the definition of the edges based on similarity, monoplex networks were fully connected by edges with different weights. The application of a cut-off value or threshold is a common practice to reduce the density of networks and find node clusters^15^. Thus, we selected a threshold (*th*) and applied it to the adjacency matrix (*A* = (*a*_*ij*_)); values in the matrix below a given threshold were removed and a new unweighted matrix (*B*) was generated (Eq. 3).

3 $$B = \left\{ {\begin{array}{*{20}c} {b_{ij} = 1, \;\;\;\;\; {a}_{ij} > th} \\ {b_{ij} = 0, \;\;\;\;\; {a}_{ij} \le th} \\ \end{array} } \right.$$

Given the possible differences in the similarity between farm AMR patterns, applying the same value for *th* would not be appropriate for each pathogen. Therefore, we assumed that the network given *th* should retain a sufficient number of edges, so there was no connected component larger than 3% of *F*_*n*_ disconnected from the main connected network. We iterated through the number of connected components based on the defined criterion and chose the highest value of *th* with only one connected component. This criterion allowed us to obtain centrality measures based on average path length, and to identify hub nodes connecting different communities^16^. The resultant network was undirected and unweighted.

For each node in the monoplex network for the included pathogens, degree, eigenvector and betweenness centrality measures were computed. These centrality measures are commonly used in network analysis and are thoroughly reviewed elsewhere^17^. Briefly, in an undirected network, the degree centrality for a node is the number of edges that connect a node with other nodes. The eigenvector centrality is based on the number of connections of a given node, as does the degree, but also takes into account the importance of its neighbors; connections to high-scoring nodes contribute more to the score of the given node^12^. Therefore, in the context of undirected similarity networks, a high degree or eigenvector score point to nodes that possess high similarity with other nodes in the network. Betweenness centrality quantifies the number of shortest paths between all node pairs passing through that node^18^.

Another important aspect to consider is the identification of communities or groups of nodes in the network and the intrinsic characteristics that differentiate them from the others. Here, we adopted the understanding of the term *“community”* as a subset of nodes in a network with more connections among themselves than with the rest of the nodes^15,19^. We used the Louvain algorithm to identify communities in the pathogen’s monoplex networks^20^. For each community, we computed the antimicrobial resistance profile by averaging the given resistance pattern of every farm in the community. Identifying communities in those networks is essential for identifying and analyzing differences in their antimicrobial resistance profiles.

Differences in the antimicrobial resistance profiles and the identification of hubs in the network based on centrality measures provide epidemiologic context for management in specific farms and suggest flows of pigs that may minimize AMR spread. The process mentioned above was developed in R version 3.6.3^21^, using the following packages: “igraph”^22^, “tidyverse”^23^, “mice”^24^, and “reshape2”^25^.

### Generation of multiplex networks

Similar to monoplex networks, multilayer networks are composed of nodes and edges, but they also add layers to their structure. Multiplex networks, also known as edge-colored networks, are a special type of multilayer network, in which the nodes are the same or similar in each layer, and are connected to their counterparts on other layers by coupling edges. As in monoplex networks, intra-layer edges connect nodes in the same layer^11,26^. Multiplex networks are valuable for studying different interactions and relationships between nodes^27^. In our study, the goal of using a multiplex network was to identify similarities among farms with information for different pathogens. Each layer would be every pathogen’s monoplex network included in the study. To assess how many nodes overlapped between layers, we compute the pairwise Jaccard similarity between nodes of the layers^19,28^. This index is equal to the number of common elements (intersection) of two sets of nodes (*L*_*i*_, *L*_*j*_) divided by the total size of the compared sets^29^ (Eq. 4). The analysis was performed using the “multinet” R package^30^.

4 $$J({L}_{i},{ L}_{j}) =\frac{({L}_{i} \cap {L}_{j})}{({L}_{i} \cup { L}_{j})}$$

## Results

### Monoplex networks

Due to the nature of the dataset, not all the tested antimicrobials were included in the laboratory panel or had consistent susceptibility interpretation criteria for each pathogen (Supplementary Fig. S1). Therefore, the size of the vector *F* varied, ranging between 12 for *E. coli* and 15 for *B. bronchiseptica* and *P. multocida*.

Overall missing information about the AMR status (susceptible or resistant) for antibiotics in the panel of a pathogen varied from 1.77% to 11.58%. However, missing information was concentrated in only some antimicrobials, varying from one for *E. coli* (tetracycline) to four for *Salmonella* spp. (Table 1). The majority of missing data was due to the lack of inclusion of an antimicrobial when reporting the results.

**Table 1:** Number of farms and associated information of the monoplex networks for each pathogen included in the study.

Pathogen	Number of farms	Overall number and percentage of missing data	Number and percentage of antibiotics with missing data	Network threshold	Network density
*Actinobacillus suis*	105	26/1470 (1.77)	2/14 (14.29)	0.8	0.312
*Bordetella bronchiseptica*	132	50/1980 (2.53)	2/15 (13.33)	0.73	0.375
*Escherichia coli*	221	107/2652 (4.03)	1/12 (8.33)	0.75	0.045
*Glaesserella parasuis*	115	108/1495 (7.22)	2/13 (15.38)	0.72	0.106
*Pasteurella multocida*	252	122/3780 (3.23)	2/15 (13.33)	0.79	0.099
*Salmonella* spp	178	268/2314 (11.58)	4/13 (30.77)	0.7	0.058
*Streptococcus suis*	309	371/4326 (8.58)	3/14 (21.43)	0.77	0.094

**Figure 1:**
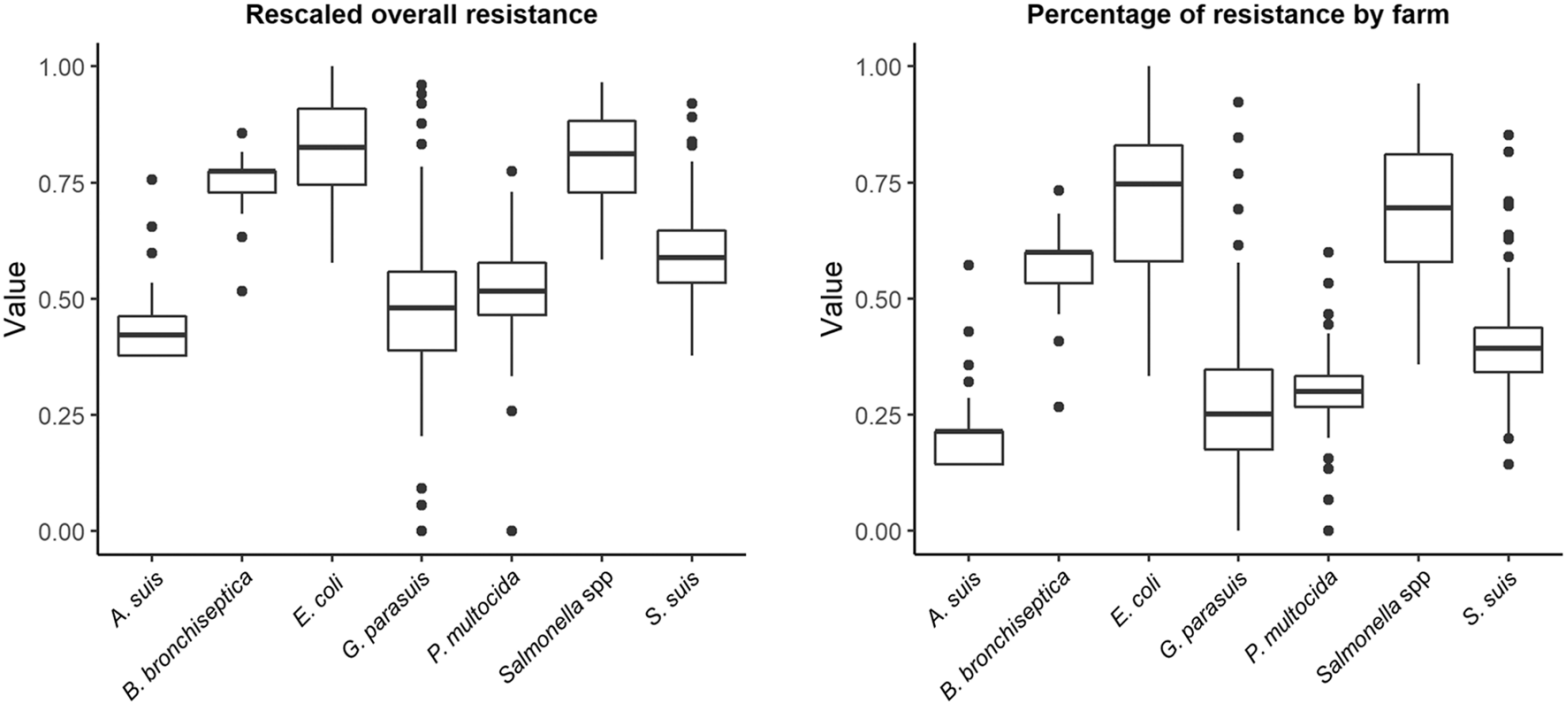
Boxplots showing the comparison of the rescaled overall resistance status for the farms (*RS*_*F*_) and the percentage of resistance by farm for the pathogens included in the study. The *RS*_*F*_ was calculated using Eq. (1) and rescaled to a maximum of 1 by dividing it by the maximal *RS*_*F*_ possible value for each pathogen. Figure generated using the “tidyverse” package in R v3.6.3.

We found significant differences in terms of the overall resistance status of the farms (*RS*_*F*_) and the percentage of resistance between pathogens (Fig. 1). The range of *RS*_*F*_ is determined by the number of included antimicrobials from the pathogen’s panel in the study due to the inclusion criteria. Since the total number of studied antimicrobials, although similar, was not the same for every pathogen, we rescaled the overall resistance status values to a maximum of 1, dividing by the maximal *RS*_*F*_ possible value for each pathogen. The small variation in the number of tested antimicrobials allowed us to easily compare them once rescaled. *E. coli* and *Salmonella* spp. isolates presented the higher AMR overall resistance while *A. suis* and *G. parasuis’* isolates the lowest, closely followed by *P. multocida*.

**Figure 2:**
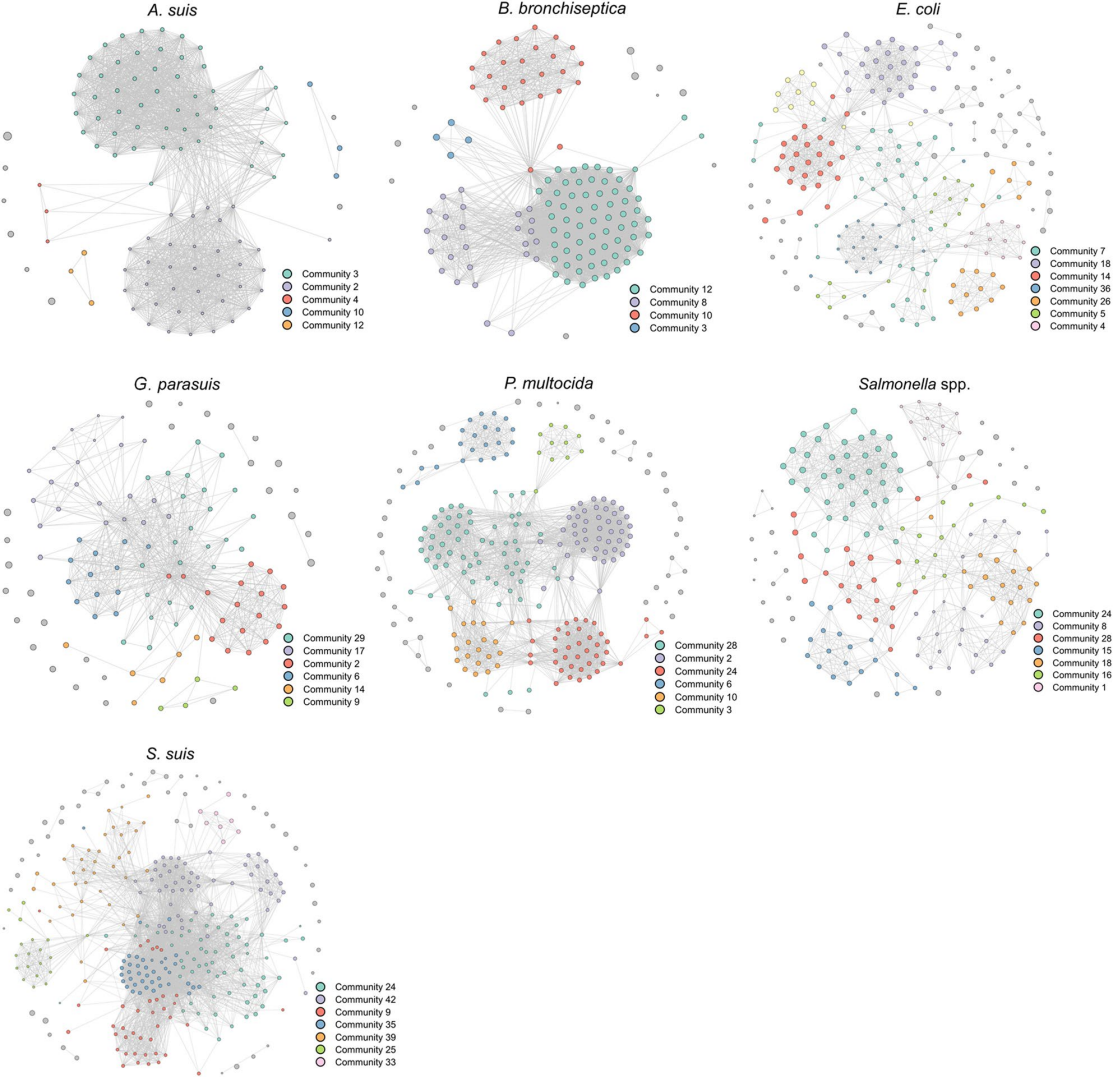
Monoplex similarity networks for each pathogen included in the study. Networks were represented using the Fruchterman–Reingold layout in Gephi^31^. In the network, node size represents the relative overall resistance status (Eq. 1), and the color the different communities identified.

**Figure 3:**
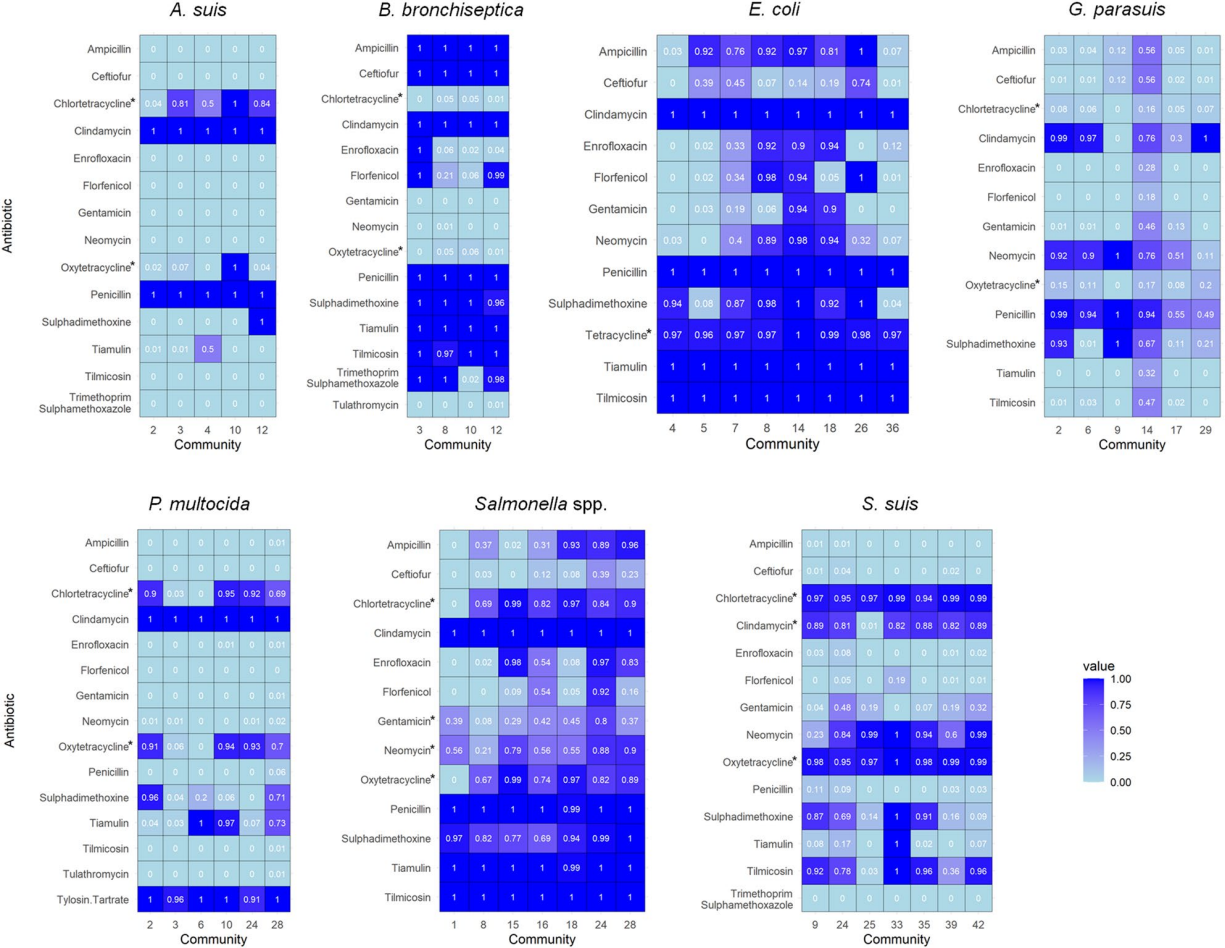
Community profile for the most important connected communities identified in the network for each pathogen included in the study. The asterisk indicates the antimicrobials for which data is missing. Figure generated using the “tidyverse” package in R v3.6.3.

The number of farms with a resistance pattern for a given pathogen varied, as did the threshold ranging between 0.7 and 0.8 (Table 1, Supplementary Fig. S2). Densities of the resultant networks are gathered in Table 1. They varied significantly for some pathogens, being as high as 0.375 for *B. bronchiseptica* and as low as 0.045 for *E. coli*. Differences in the morphology and density of the networks for the different pathogens studied here can be observed (Fig. 2). Thus, the number of identified communities by the Louvain algorithm also varied from highly dense networks to less dense ones. Single-member communities were considered as isolates. Networks for *A. suis* and *B. bronchiseptica* had a high density, meaning that the isolates tested in the farms had extremely similar patterns of resistance, and therefore fewer communities were identified. AMR profiles were similar among communities for these pathogens (Fig. 3). In both cases, more than 48% of the farms belonged to a single community. *E. coli* and *Salmonella* spp. are pathogens with higher overall resistance in this study (Fig. 1), and that is also reflected in their community profiles (Fig. 3).

**Figure 4:**
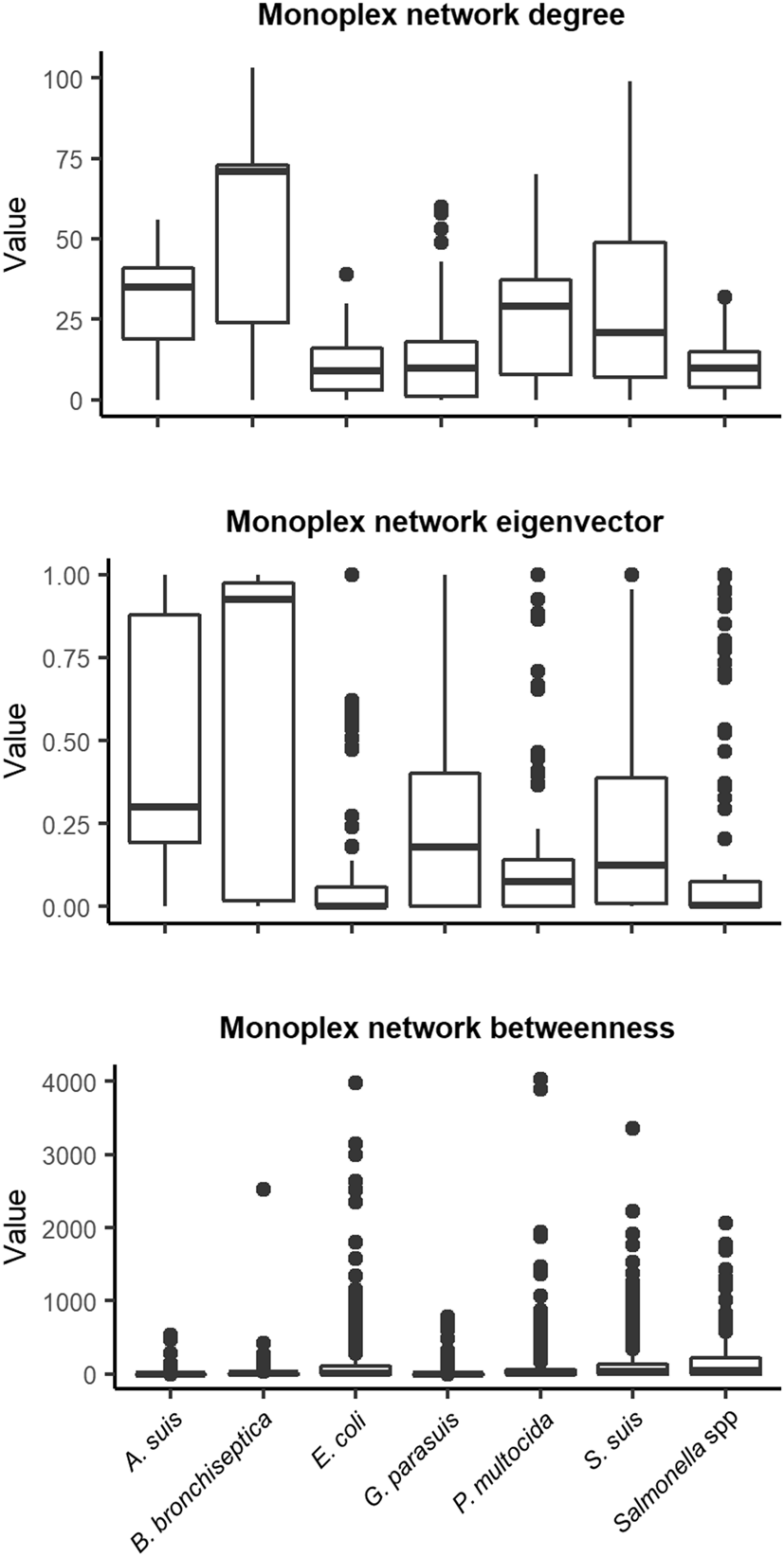
Network’s centrality measures for each pathogen included in the study. Figure generated using the “tidyverse” package in R v3.6.3.

Several centrality measures were also computed, namely degree, eigenvector and betweenness centralities (Fig. 4). These measures allowed us to identify nodes that are hubs in the network^17^. Higher values for degree centrality showed farms that have more similarity to other farms, while the eigenvector showed farms that are connected to other well-connected farms. Higher values of betweenness showed hub farms that connect different communities, and therefore could be individually analyzed for their implication in AMR spread. It can be seen how denser networks, such as the ones for *A. suis* and *B. bronchiseptica*, have in general greater degree and eigenvector values and fewer betweenness hubs.

### Multiplex networks

**Figure 5:**
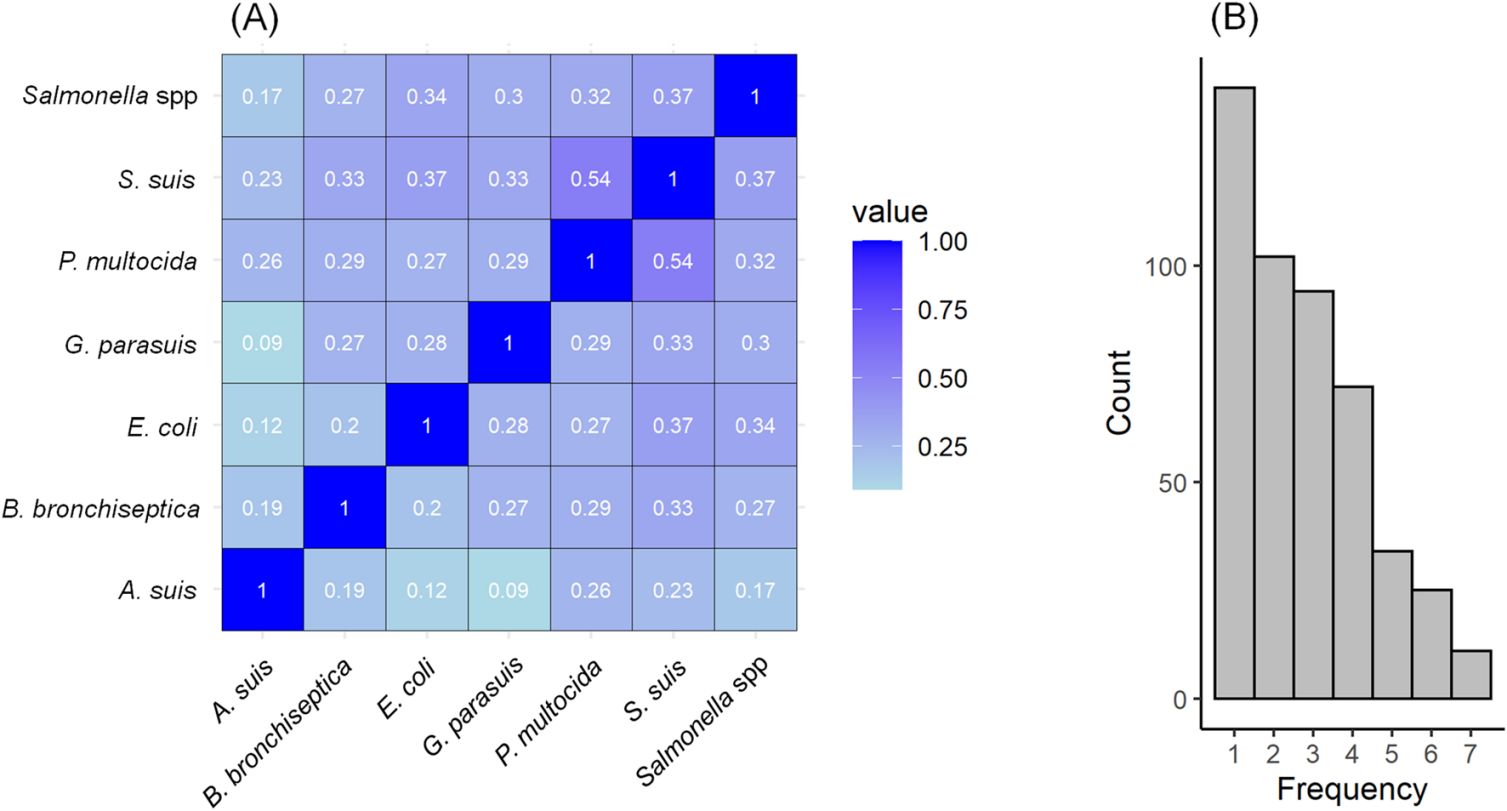
(**A**) Jaccard similarity of the shared nodes of every pair of layers, and (**B**) histogram showing the frequency of presence of farms in the multiplex network. Figure generated using the “tidyverse” package in R v3.6.3.

We generated a multiplex network that was comprised of 479 nodes, although not all the farms had information about every pathogen (Fig. 5). Many of them had information about only one pathogen (Fig. 5B). According to the Jaccard similarity, the overlapping of nodes between layers is low, being inferior to 0.55, and it is the lowest for *A. suis* with the rest of the pathogen layers (Fig. 5A).

## Discussion

In this study, we propose a novel approach of using similarity networks and community detection to generate AMR profiles as a quick and easy visualization tool to support decision-making and improve antimicrobial stewardship for swine production systems. The pathogens studied in this work are etiological agents of important systemic, enteric, and respiratory diseases in swine, which create a significant impact on the health and well-being of pigs, as well as an important economic loss for the swine industry. Furthermore, these agents are primary drivers of antimicrobial use on farms. There is also a public health concern about some of these agents, such as *E. coli*, *Pasteurella multocida*, *Salmonella *spp., *S. suis* and *B. bronchiseptica*, due to their zoonotic nature^32^. Additional information on their epidemiology, pathogenesis and control can be found elsewhere^32^.

Here, farms have been grouped in terms of their phenotypic AMR similarity. It is worth noting that *in vitro* characterization of the susceptibility of an isolate does not necessarily imply the success or failure of the *in vivo* clinical outcome^33^. There are some uncertainties of clinical breakpoints in veterinary medicine for many antimicrobials since they are host-specific and even vary for different target organs^34,35^. In this study, we have used the currently approved approach for assigning the phenotypic AMR status based on CLSI veterinary guidelines and laboratory expertise that are therapeutically relevant^10^. Moreover, these clinical breakpoints were used to show the AMR profile of the bacterial population of the farm and are epidemiological relevant for the current study regardless of the sampled organ. The phenotypic AMR similarity, along with the AMR profile of the identified communities, can help improve the therapeutic management of farms, as well as guide animal movements between them to reduce the spread of resistant clones. For example, in the case of *S. suis*, the most frequent antimicrobial treatments are beta-lactams and fluoroquinolones^36^, so resistance to one of these antimicrobials would be highly relevant for infection management, and its spread could be mitigated by large swine production systems and veterinarian practitioners with the information provided by the methodology proposed here. In the *S. suis* community profile (Fig. 3), it can be seen that the major communities are highly susceptible to those antimicrobials. Still, if susceptibility to one of them decreases over time, specific and localized management measures could be taken to hamper the spread of resistance.

For some farms, only information about the susceptibility profile of one isolate was available. Thus, farm patterns should not be considered as the AMR frequency of the entire farm, since only one isolate per farm was used for the generation of the network in those cases. Therefore, for those farms, the information presented here should be considered as a relative risk of resistance and not as the overall resistance of the farm. In addition, there might be a possible bias in the data collection since, in some cases, sampling was clinically based and did not proceed from random sampling. This sampling criteria along with the consideration of the AMR’s categories of “resistance” and “intermediate” status as resistant isolates, might increase the percentage of resistance found for each pathogen for some antimicrobials that will be discussed below. For future studies and to minimize the former limitation, sampling protocols could be implemented. This protocol should include how to calculate sample size so it is representative of the farm’s population in terms of age, sex and number of animals, as well as to obtain an appropriate estimate of the prevalence of AMR^37–39^. Sampling should be carried out preferably randomly in healthy animals whenever possible, although the inclusion of samples from sick animals could be accepted in an admissible proportion in the shake of cost-effectiveness. Moreover, guidelines can be provided as to the target organs and type of sample admissible to ensure comparable results^37,38^. Even considering these limitations, the methodology is valuable from a practical point of view for identifying similarity patterns between farms affected by important pathogens of animal and public health concern.

One example of applicability is minimizing animal flows from farms belonging to other communities whenever possible; and even more so from communities with higher resistance status or where a pathogen is resistant to a therapeutically crucial antimicrobial. This could be even more beneficial when resistance genes are evaluated. The animal source may be outside the network, whether a farm that has not been tested or a farm for which no information is available for a given pathogen. In that case, an analysis of the AMR profiles of recipient farms can be conducted to find similarities between them, which could include genetic factors as well. Unfortunately, we could not assess shared features between farms, such as the geographical location and type of farm, flow or origin of pigs, farm size, treatment, or biosecurity protocols, among other factors. For example, the type of farm could be associated with different management practices^7^. This information would be highly beneficial to identify whether there are common features that might be contributing to increasing AMR, facilitating the benchmarking of farms and supporting the implementation of mitigation strategies. Furthermore, having information about the community’s profile can enhance the application of an effective clinical treatment in the early stages of an outbreak, and, therefore, better inform antimicrobial use and avoid the excessive use or misuse of antibiotics that could contribute to AMR. Therefore, this tool should be considered as a preliminary approach to finding meaningful associations between antimicrobial patterns of farms that can help tackle the AMR problem.

Regarding methodological aspects, missing values are a frequent problem in any analysis^14^. The primary source of missing data was for tetracyclines, since some laboratories reported only tetracycline, while others included chlortetracycline and oxytetracycline, but not the three of them combined. Hence, information about the resistance profile of certain antimicrobials should be evaluated taking into consideration the need for imputation for certain data. However, the imputation of missing data allows the use and analysis of valuable partial information that would not be included otherwise^14^. In this study, we kept phenotypic AMR data in the farm’s antimicrobial pattern even with < 50% of missing information for some pathogens, so that we could compare such resistance profiles with other pathogens, and also as a way to highlight the importance of having complete information about the same AMR panel. Community profiles for *A. suis* and *P. multocida* are a prime example of the importance of keeping phenotypic AMR for an antimicrobial with missing information, since for several of them, significant differences in community profiles could be revealed. In both cases, we identified communities that were highly susceptible to chlortetracycline: community 2 for *A. suis* and communities 3 and 6 for *P. multocida*. Something similar occurred with *S. suis* network and clindamycin susceptibility for the different communities. Community 25 had 1% resistant bacteria while the rest of the large communities exceeded 80% of resistance.

The reason for using the average of the available information of other farms for the same pathogen is that it shows the probability of resistance in a farm with missing information. Although it is a limitation of the study, we believe this is a simple but effective approach for this situation because it maintains the characteristics of the dataset and prevents the loss of data. Moreover, other imputation methods, such as predictive mean matching would assign a resistant or susceptible status^24^, directly distorting the similarity networks by potentially grouping farms with missing data with resistant or susceptible groups, when their real status is unknown for a given antimicrobial.

The use of a threshold is essential when working with fully connected networks to reduce their density and properly analyze meaningful node relationships. In many studies facing the same issue, researchers defined this threshold by trial and error^40^, although some threshold selection methods have been described in the literature^41^. We have developed a general threshold selection method that allows us to automatically create a similarity network that retains valuable information about path-length centrality measures, such as betweenness. As our results showed (Table 1), giving the same threshold for every pathogen would be arbitrary and unappropriated due to the specific intrinsic characteristics of each phenotypic AMR similarity network.

The multiplex network could not be adequately generated due to the lack of critical farm’ information about the AMR patterns for some pathogens (Fig. 5). Considering the small overlapping of nodes observed between layers, centrality measures would have just highlighted farms that appeared in more layers, and not necessarily the most important ones. A multiplex approach could be applied when more AMR data is available in all or at least in most farms. This would allow for the identification of farms that share the same AMR similarity pattern for the different pathogens. Similarity networks can be combined with other networks, such as networks representing the movement of animals between farms or other features of interest. Another application would be the generation of temporal networks to analyze the progress and evolution of relationships of AMR for a particular pathogen in a specified period^11^. However, temporal data about AMR in each farm is required, and this information was limited in this study (i.e., the range of farms with a single isolate ranged from 56.63% for *S. suis* to 78.1% for *A. suis*).

Although not the aim of the study, we discuss below the AMR frequency for the different pathogens and its impact based on the most relevant antimicrobials used for the clinical treatment of each pathogen. It should be noted that AMR frequency among pathogens is difficult to compare between studies due to differences in study design, the target population, as well as resistance criteria and clinical breakpoint^35^.

Therapeutic options to treat the disease caused by the infection of *A. suis* include amoxicillin, ampicillin, penicillin, tiamulin, ceftiofur, gentamicin and trimethoprim/sulfadiazine^42^. Taking this into account, *A. suis* isolates from the major communities of this study are susceptible to many of the therapeutic tools against this pathogen (Figs. 2 and 3).

Similar to previous reports, almost all isolates of *B. bronchiseptica* were resistant to beta-lactams (99% here vs. 100%)^43^, which are commonly used for the treatment of swine respiratory disease^44^. The resistance to beta-lactams is explained by a species-specific beta-lactamase gene described for the bacteria, as well as low membrane permeability^45^. Almost all isolates were susceptible to tulathromycin, an antibiotic used for the treatment of swine respiratory disease that comprises *Actinobacillus pleuropneumoniae*, *P. multocida*, *Mycoplasma hyopneumoniae*, *G. parasuis* and *B. bronchiseptica*^46^. *B. bronchiseptica* isolates were largely susceptible to enrofloxacin^44^, although community 3 was phenotypically resistant. Differences in florfenicol susceptibility have been described in several studies ranging from 10 to almost 98%^45^. In accordance with those results, we observed significant differences between communities (Fig. 3).

*P. multocida* can be found co-infecting with *B. bronchiseptica* in the swine upper respiratory tract, so phenotypic susceptibility of both pathogens should be taken into account in the antimicrobial treatment^44,47^. *P. multocida* isolates presented more overall susceptibility to the tested antimicrobials than other pathogens (Fig. 1), although nearly all isolates were phenotypically resistant to clindamycin and tylosin-tartrate. This elevated susceptibility status for most isolates is in agreement with the majority of published work^35^. Drugs commonly used to treat the disease caused by this bacteria are ampicillin, ceftiofur, enrofloxacin, and tulathromycin^47^, for which the majority of isolates were susceptible (Fig. 3).

There are no international criteria for *G. parasuis* clinical breakpoints, so the comparison between studies is challenging^35^. In Dayao et al., isolates of *G. parasuis* had higher MIC values for ampicillin, penicillin and tetracycline^48^. Similarly, in this study, the majority of large communities showed elevated resistance to penicillin.

The phenotypic resistance results obtained here for *S. suis* are in agreement with other studies. In general, low resistance was observed for beta-lactams, and high resistance was observed for tetracyclines^35,36,49^. In several countries, high levels of resistance to macrolides and lincosamides have been reported^49^, although, in this study, some communities were highly susceptible to these drugs. Moreover, *S. suis* was highly susceptible to florfenicol in the study farms, an observation also reported in European and North American countries^35^.

*Escherichia coli* was recently considered, alongside *Brachyspira hyodysenteriae*, to be the most critical antimicrobial-resistant bacteria in the European Union for swine^35^. In a temporal study conducted in the USA, they found high levels of resistance to tetracyclines and ampicillin, as well as moderate levels of resistance to gentamicin, neomycin, and sulfonamides^50^. Similar to the present study, Jiang et al. reported elevated AMR in *E. coli* isolates from the intestinal contents or fecal samples of diarrheic piglets from 15 states of the United States, where all isolates were found to be resistant to clindamycin, penicillin, tiamulin, and tilmicosin. In addition, they observed high resistance to ampicillin, chlortetracycline, oxytetracycline and sulphadimethoxine^51^. It is also worth noting that Gram-negative bacteria, for instance, *E. coli*, possess relative intrinsic resistance to some antimicrobial classes such as macrolides^52^, which is consistent with our data.

*In vitro* resistance to multiple drugs is often observed for species of the genus *Salmonella.* Therefore, the assessment of AMR prior to the onset of a therapeutic solution is essential^53^. Multidrug resistance bacteria constitute a problem for effective clinical treatment of infections. Therefore, for pathogens that have developed resistance to several drugs, knowing the historical resistance of a population can serve as a useful guide to assessing therapeutic options in the early stages of an outbreak^53,54^.

Given the importance of the AMR problem, developing new strategies is paramount to properly address and mitigate it in livestock husbandry in the context of the One Health approach. The methodology, analysis, and visualization presented here are highly relevant, not only to guide more effective clinical actions in a given farm, but to enhance antimicrobial stewardship and decrease the spread of resistance of key antimicrobials across farms, as exemplified in the discussion. This approach can be easily expanded and enhanced to incorporate animal, farm and system features to better understand their impact on AMR patterns and further support the implementation of preventive measures to reduce AMR spread. We have also highlighted the potential key role of high-betweenness farms in the different networks that connect communities. The characteristics of these farms should be analyzed in depth for their implication in AMR spread.

## Supplementary Information

Supplementary Information.

## Data Availability

The datasets analyzed during the current study are not publicly available due to confidentiality reasons and restrictions on the availability of these data, but are available from the corresponding author on reasonable request and with permission of the data provider.

## References

[1] Bright-Ponte SJ (2019). One Health and antimicrobial resistance, a United States perspective. Rev. Sci. Tech..

[2] OIE. *The OIE Strategy on Antimicrobial Resistance and the Prudent Use of Antimicrobials*, https://www.oie.int/app/uploads/2021/03/en-oie-amrstrategy.pdf (2016).

[3] WHO. *Global Action Plan On Antimicrobial Resistance*, https://ahpsr.who.int/publications/i/item/global-action-plan-on-antimicrobial-resistance (2015)

[4] CDC. *Antibiotic Resistance Threats In The United States*, https://www.cdc.gov/drugresistance/pdf/threats-report/2019-ar-threats-report-508.pdf (2019).

[5] Shallcross LJ, Howard SJ, Fowler T, Davies SC (2015). Tackling the threat of antimicrobial resistance: From policy to sustainable action. Philos. Trans. R Soc. Lond. B Biol. Sci..

[6] Bengtsson B, Greko C (2014). Antibiotic resistance—Consequences for animal health, welfare, and food production. Ups. J. Med. Sci..

[7] Wall B (2016). Drivers, dynamics and epidemiology of antimicrobial resistance in animal production.

[8] Hernando-Amado S, Coque TM, Baquero F, Martínez JL (2019). Defining and combating antibiotic resistance from One Health and Global Health perspectives. Nat. Microbiol..

[9] Pehrsson EC (2016). Interconnected microbiomes and resistomes in low-income human habitats. Nature.

[10] CLSI. *Performance Standards for Antimicrobial Disk and Dilution Susceptibility Tests for Bacteria Isolated From Animals*. 5th edn, (Clinical and Laboratory Standards Institute, 2020).

[11] Kivelä M (2014). Multilayer networks. J. Complex Netw..

[12] Ruhnau B (2000). Eigenvector-centrality—A node-centrality?. Soc. Netw..

[13] Tabak, J. Differential geometry in *Geometry: The Language of Space and Form* 248 (Facts On File, Inc, 2014).

[14] Graham, J. W., Cumsille, P. E. & Shevock, A. E. Methods for Handling Missing Data. in *Handbook of Psychology: Research methods in psychology* (ed. Schinka, J. A., W. F. Velicer, & I. B. Weiner) 109–141 (John Wiley & Sons, Inc, 2013).

[15] Fornito A, Zalesky A, Bullmore E (2016). Fundamentals of brain network analysis.

[16] Valavanis I, Spyrou G, Nikita K (2010). A similarity network approach for the analysis and comparison of protein sequence/structure sets. J. Biomed. Inform..

[17] Martínez-López B, Perez AM, Sánchez-Vizcaíno JM (2009). Social network analysis. Review of general concepts and use in preventive veterinary medicine. Transbound. Emerg. Dis..

[18] Freeman LC (1977). A set of measures of centrality based on betweenness. Sociometry.

[19] Magnani M, Rossi L, Vega D (2021). Analysis of multiplex social networks with R. J. Stat. Softw..

[20] Blondel VD, Guillaume J-L, Lambiotte R, Lefebvre E (2008). Fast unfolding of communities in large networks. J. Stat. Mech. Theory Exp..

[21] R Core Team. R: A Language and Environment for Statistical Computing. *R Foundation for Statistical Computing: Vienna, Austria* (2020).

[22] Csardi, G. & Nepusz, T. *The igraph software package for complex network research*, https://igraph.org. (2006).

[23] Wickham H (2019). Welcome to the Tidyverse. J. Open Source Softw..

[24] van Buuren S, Groothuis-Oudshoorn K (2011). mice: Multivariate imputation by chained equations in R. J. Stat. Softw..

[25] Wickham H (2007). Reshaping data with the reshape package. J. Stat. Softw..

[26] De Domenico M (2013). Mathematical formulation of multilayer networks. Phys. Rev. X.

[27] Finn KR, Silk MJ, Porter MA, Pinter-Wollman N (2019). The use of multilayer network analysis in animal behaviour. Anim. Behav..

[28] Bródka P, Chmiel A, Magnani M, Ragozini G (2018). Quantifying layer similarity in multiplex networks: A systematic study. R. Soc. Open. Sci..

[29] Jaccard P (1912). The distribution of the flora of the alpine zone. New Phytol..

[30] Magnani, M., Rossi, L., Hanteer, O., Vega, D. & Dubik, M. *multinet: Analysis and Mining of Multilayer Social Networks*, https://CRAN.R-project.org/package=multinet (2021).

[31] Bastian, M., Heymann, S. & Jacomy, M. Gephi: an open source software for exploring and manipulating networks. in *3rd International AAAI Conference on Weblogs and Social Media.* (2009).

[32] Zimmerman J (2019). Diseases of Swine.

[33] Leekha S, Terrell CL, Edson RS (2011). General principles of antimicrobial therapy. Mayo Clin. Proc..

[34] Aarestrup FM, Oliver Duran C, Burch DG (2008). Antimicrobial resistance in swine production. Anim. Health Res. Rev..

[35] EFSA Panel on Animal Health and Welfare (AHAW) (2021). Scientific Opinion on the assessment of animal diseases caused by bacteria resistant to antimicrobials: Swine. EFSA J..

[36] Seitz M, Valentin-Weigand P, Willenborg J (2016). Use of antibiotics and antimicrobial resistance in veterinary medicine as exemplified by the swine pathogen *Streptococcus suis*. Curr. Top Microbiol. Immunol..

[37] WHO. *Integrated surveillance of antimicrobial resistance in foodborne bacteria: application of a one health approach: guidance from the WHO Advisory Group on Integrated Surveillanec of Antimicrobial Resistance (AGISAR)*. 88 p. (World Health Organization, 2017).

[38] WOA H. Chapter 6.7: Harmonisation of national antimicrobial resistance surveillance and monitoring programmes. in *Terrestrial Animal Health Code* (ed World Organisation for Animal Health) (2022).

[39] Cochran WG (1977). Sampling Techniques.

[40] Catanese HN, Brayton KA, Gebremedhin AH (2018). A nearest-neighbors network model for sequence data reveals new insight into genotype distribution of a pathogen. BMC Bioinform..

[41] Apeltsin L, Morris JH, Babbitt PC, Ferrin TE (2011). Improving the quality of protein similarity network clustering algorithms using the network edge weight distribution. Bioinformatics.

[42] Gottschalk, M. & Broes, A. Actinobacillosis. in *Diseases of Swine* (ed. Zimmerman, J. J. *et al.*) 749–766 (2019).

[43] Dayao DA, Gibson JS, Blackall PJ, Turni C (2014). Antimicrobial resistance in bacteria associated with porcine respiratory disease in Australia. Vet. Microbiol..

[44] Brockmeier, S. L., Register, K. B., Nicholson, T. L. & Loving, C. L. Bordetellosis. in *Diseases of Swine* (ed. Zimmerman, J. J. *et al.*) 767–777 (2019).

[45] Kadlec K, Schwarz S (2018). Antimicrobial Resistance in Bordetella bronchiseptica. Microbiol. Spectr..

[46] EMA. *EPAR summary for the public: Draxxin*, https://www.ema.europa.eu/en/documents/overview/draxxin-epar-summary-public_en.pdf (2016).

[47] Register, K. B. & Brockmeier, S. L. Pasteurellosis. in *Diseases of Swine* (ed. Zimmerman, J. J. *et al.*) 884–897 (2019).

[48] Dayao D, Gibson JS, Blackall PJ, Turni C (2016). Antimicrobial resistance genes in *Actinobacillus pleuropneumoniae*, *Haemophilus parasuis* and *Pasteurella multocida* isolated from Australian pigs. Aust. Vet. J..

[49] Varela NP (2013). Antimicrobial resistance and prudent drug use for *Streptococcus suis*. Anim. Health Res. Rev..

[50] Malik YS, Chander Y, Olsen K, Goyal SM (2011). Antimicrobial resistance in enteric pathogens isolated from Minnesota pigs from 1995 to 2004. Can. J. Vet. Res..

[51] Jiang F (2019). Genotypes and antimicrobial susceptibility profiles of hemolytic *escherichia coli* from diarrheic piglets. Foodborne Pathog. Dis..

[52] Cox G, Wright GD (2013). Intrinsic antibiotic resistance: Mechanisms, origins, challenges and solutions. Int. J. Med. Microbiol..

[53] Griffith, R. W., Carlson, S. A. & Krull, A. C. Salmonellosis. in *Diseases of Swine* (ed. Zimmerman, J. J. *et al.*) 912–925 (2019).

[54] Fairbrother, J. M. & Nadeau, É. Colibacillosis. in *Diseases of Swine* (ed. Zimmerman, J. J. *et al.*) 807–834 (2019).

