# Supplementary material for: A tool to enhance antimicrobial stewardship using similarity networks to identify antimicrobial resistance patterns across farms
Source: Sci Rep. 2023 Feb 20;13:2931. doi: 10.1038/s41598-023-29980-4 (PMC9941107; doi:10.1038/s41598-023-29980-4)
Supplement: Supplementary file 1 — Supplementary Information. [file 41598_2023_29980_MOESM1_ESM.pdf]

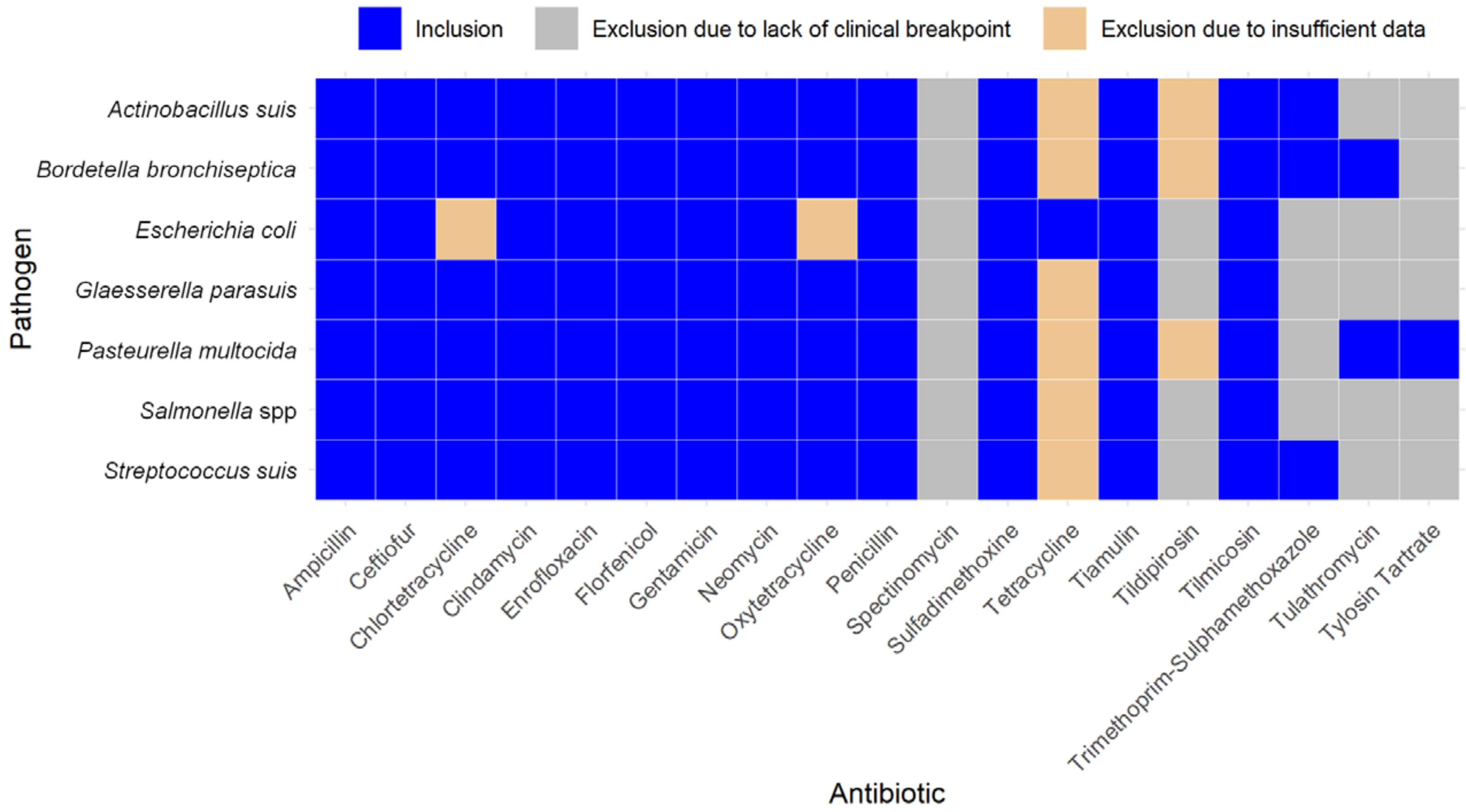

Supplementary Figure S1. Included and excluded antibiotics of the panel for each pathogen studied.

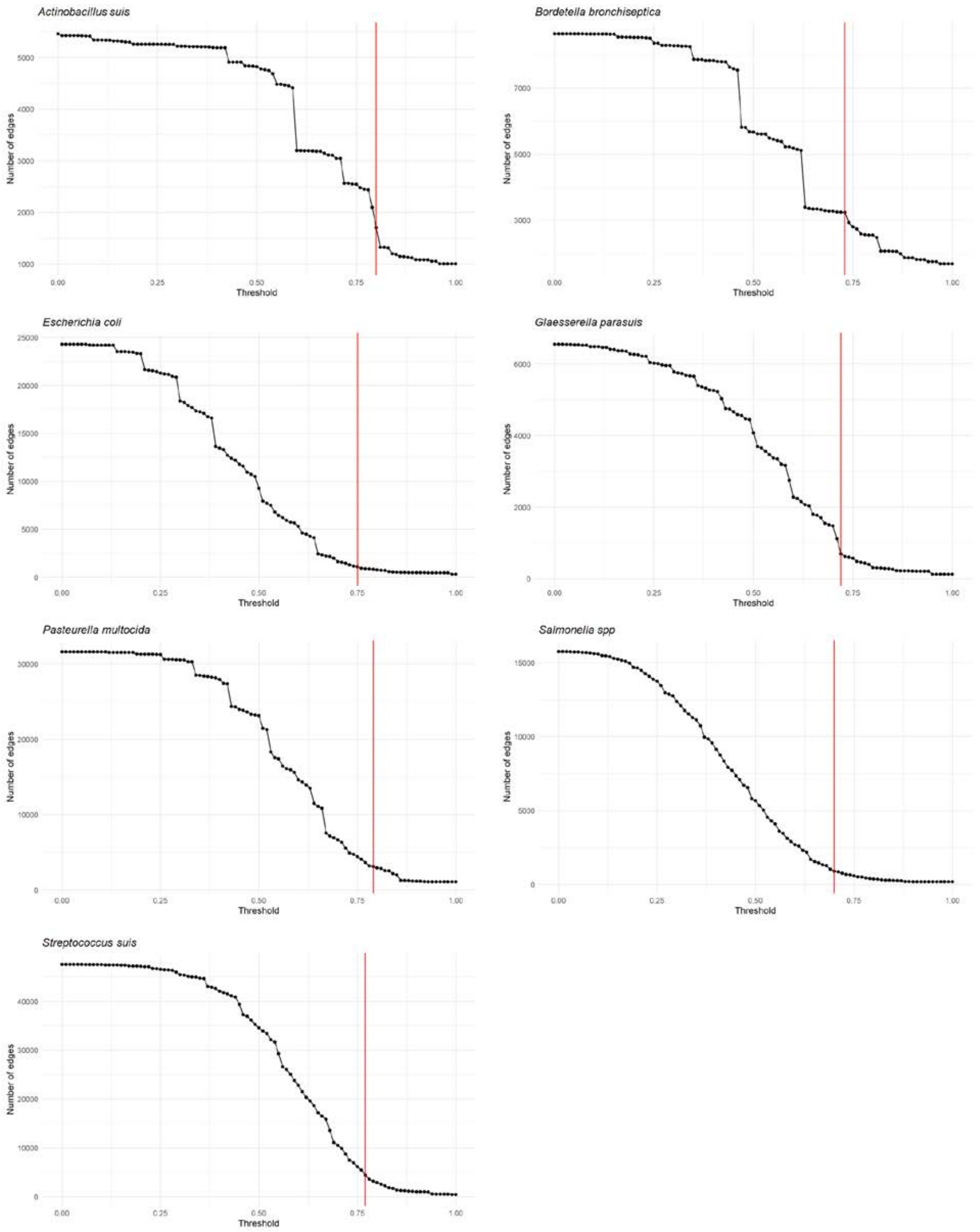

Supplementary Figure S2. Number of edges in the monoplex similarity networks for every threshold and each pathogen included in the study. The vertical red line indicates the selected threshold.
